# PECSS: Pulmonary Embolism Comprehensive Screening Score to safely rule out pulmonary embolism among suspected patients presenting to emergency department

**DOI:** 10.1186/s12890-023-02580-8

**Published:** 2023-08-07

**Authors:** Luojia Tang, Yundi Hu, Dong Pan, Chun Yang, Cheng Tang, Yunchuan Huang, Jianyong Gu, Min Min, Xiaolei Lin, Chaoyang Tong

**Affiliations:** 1 Emergency Department of Zhongshan Hospital, Fudan University, Shanghai, China; 2 School of Data Science, Fudan University, Shanghai, China; 3 Department of Information and Intelligence Development of Zhongshan Hospital, Fudan University, Shanghai, China

**Keywords:** Pulmonary Embolism, Pretest probability, Emergency Department, D-Dimer, Screening Score

## Abstract

**Background:**

Pulmonary embolism is a severe cardiovascular disease and can be life-threatening if left untreated. However, the detection rate of pulmonary embolism using existing pretest probability scores remained relatively low and clinical rule out often relied on excessive use of computed tomographic pulmonary angiography.

**Methods:**

We retrospectively collected data from pulmonary embolism suspected patients in Zhongshan Hospital from July 2018 to October 2022. Pulmonary embolism diagnosis and severity grades were confirmed by computed tomographic pulmonary angiography. Patients were randomly divided into derivation and validation set. To construct the Pulmonary Embolism Comprehensive Screening Score (PECSS), we first screened for candidate clinical predictors using univariate logistic regression models. These predictors were then included in a searching algorithm with indicators of Wells score, where a series of points were assigned to each predictor. Optimal D-Dimer cutoff values were investigated and incorporated with PECSS to rule out pulmonary embolism.

**Results:**

In addition to Wells score, PECSS identified seven clinical predictors (anhelation, abnormal blood pressure, in critical condition when admitted, age > 65 years and high levels of pro-BNP, CRP and UA,) strongly associated with pulmonary embolism. Patients can be safely ruled out of pulmonary embolism if PECSS ≤ 4, or if 4 < PECSS ≤ 6 and D-Dimer ≤ 2.5 mg/L. Comparing with Wells approach, PECSS achieved lower failure rates across all pulmonary embolism severity grades. These findings were validated in the held-out validation set.

**Conclusions:**

Compared to Wells score, PECSS approaches achieved lower failure rates and better compromise between sensitivity and specificity. Calculation of PECSS is easy and all predictors are readily available upon emergency department admission, making it widely applicable in clinical settings.

**Trail registration:**

The study was retrospectively registered (No. CJ0647) and approved by Human Genetic Resources in China in April 2022. Ethical approval was received from the Medical Ethics Committee of Zhongshan Hospital (NO.B2021-839R).

**Supplementary Information:**

The online version contains supplementary material available at 10.1186/s12890-023-02580-8.

## Background

Pulmonary embolism (PE) is a common severe cardiovascular illness andcan be life-threatening if left untreated [1–3]. However, clinical signs and symptoms have relatively low sensitivity and specificity in PE diagnosis [4, 5]. Current diagnosis of PE still relies on computed tomographic pulmonary angiography (CTPA), which is costly, time-consuming and exposes patients to extra radiation [6, 7]. Recent studies in North America showed that the detection rate of suspected PE is as low as 5% [8]. Therefore, it is of great importance to avoid excessive CTPA by considering ruling out PE using clinical pretest probability scores.

Commonly used clinical pretest probability, such as Wells score and Geneva score, combines clinical symptoms with predisposing risk factors of venous thromboembolism and classifies patients with suspected PE into distinct risk categories [9–11]. In addition to clinical pretest probability score, plasma level of D-Dimer, which is often elevated in the presence of acute thrombosis due to simultaneous activation of coagulation and fibrinolysis, is also used to rule out PE [12, 13]. The combination of low pretest probability score and plasma D-Dimer level lower than 500 ug/L were used to rule out PE in an initial attempt, followed by age adjusted D-Dimer cutoff (500 ug/L for patients below 50 years, and age*10 ug/L for patients older than 50 years) and risk category specific D-Dimer cutoffs (1000 ug/L for patients with low pretest score, and 500 ug/L for patients with moderate pretest score) [14–16]. Despite efforts in adjusting D-Dimer thresholds in different age groups and PE risk categories, specificities and negative predictive values of the PE rule out strategies are generally low, resulting in an overuse of CTPA among alternatively diagnosed patients with high plasma D-Dimer levels or moderate pretest probability score. What’s more, none of the current PE rule out strategies differentiate the failure rates across different PE severity grades, as major embolism and multiple embolisms require much more stringent diagnosis and treatment window than mild embolism [17].

In this investigation, we aim to develop a comprehensive PE pretest score that incorporates clinical indications in Wells score and other important clinical predictors to guide clinicians in assessing PE risk with improved screening performances. The proposed PE Comprehensive Screening Score (PECSS) is designed to optimize the rule out performance among patients with severe PE and to achieve excellent operating characteristics among patients with moderate and mild PE. PE severity grades were evaluated based on CTPA and validation of PECSS was conducted in an independent held-out data set.

## Methods

### Data collection

We retrospectively collected data from patients presenting to the emergency department of Zhongshan Hospital (affiliated to Fudan University) from July 2018 to October 2022. Patients were included if they were 18 years and older, were suspected of PE or could not be ruled out of PE by the attending physicians. Patients were excluded if they did not have PE status or severity confirmed by CTPA, had missing D-Dimer values and were pregnant. Electronic medical records, CTPA, laboratory tests results and vital signs were collected and reviewed.

### PE severity grade

The severity grade of PE was determined based on CTPA. PE was divided into 4 severity grade categories: 1) severe PE, which was defined as the pulmonary artery embolism or multiple pulmonary arteriole embolism; 2) moderate PE, defined as pulmonary artery branch embolism; 3) mild PE, defined as single pulmonary arteriole embolism; 4) no PE.

### Study design

Patients meeting the inclusion and exclusion criteria were randomly divided into the derivation (80%) and validation (20%) set according to the stratified randomization scheme such that the distribution of PE severity grades were similar in the two data sets. Derivation set was used to construct the new screening approach and compare it to existing approaches, while the validation set was used to validate the findings. The primary endpoint was failure rate in patients with severe PE, which was defined as the proportion of PE cases failed to be ruled out in patients with severe PE. Secondary endpoints include failure rate in moderate, mild and total PE, defined as the proportion of PE cases failed to be ruled out in patients with moderate, mild and any PE, respectively. In addition, we also evaluated the sensitivity, specificity, positive predictive value (PPV) and negative predictive value (NPV) associated with each screening approach.

### Statistical analysis

Statistical analysis was performed using R statistical software version 4.0.3. Continuous characteristics were described using mean $\pm$ standard deviation if normally distributed, or median (interquartile range) if normality was violated. Categorical characteristics were presented using frequency (percentage) per category. Statistical tests were performed using student t test or ANOVA for continuous variables (Wilcoxon rank sum test for skewed distribution), and Chi-square or Fisher’s exact test for categorical variables.

To construct the PE comprehensive screening score (PECSS), we first screened all 26 clinical predictors that were suspected to be associated with PE according to clinical consensus. Among all these predictors, Lactic Acid, Aspartate Transaminase and Lactic Dehydrogenase had missing proportions higher than 30% and were therefore dropped from the following analyses. Next, univariate association with PE status (PE vs no PE) of each predictor was assessed using univariate logistic regression model, and predictors with P values smaller than 0.05 were selected as candidate variables in models constructing PECSS. Candidate variables selected by univariate logistic regression models were then included in a searching algorithm, where a series of points were assigned to each predictor and the total points were added to the Wells score to construct PECSS. To facilitate clinical practice and in line with total Wells score, we considered 0, 0.5, 1.0 and 1.5 points for each candidate variable so that higher scores indicate higher probability of PE. Finally, optimal PECSS cutoffs and optimal D-Dimer cutoffs within each PECSS stratum were determined to minimize the failure rate in patients with severe PE.

Screening performances of PECSS, PECSS + D-Dimer and PECSS + PERC were evaluated and compared to Wells score, Wells score + D-Dimer and Wells score + PERC. Failure rates among patients with severe, moderate, mild and any PE were compared under these scoring systems, respectively. Sensitivity, specificity, PPV and NPV were also calculated for each screening approach.

## Results

### Patient characteristics

**Fig. 1:**
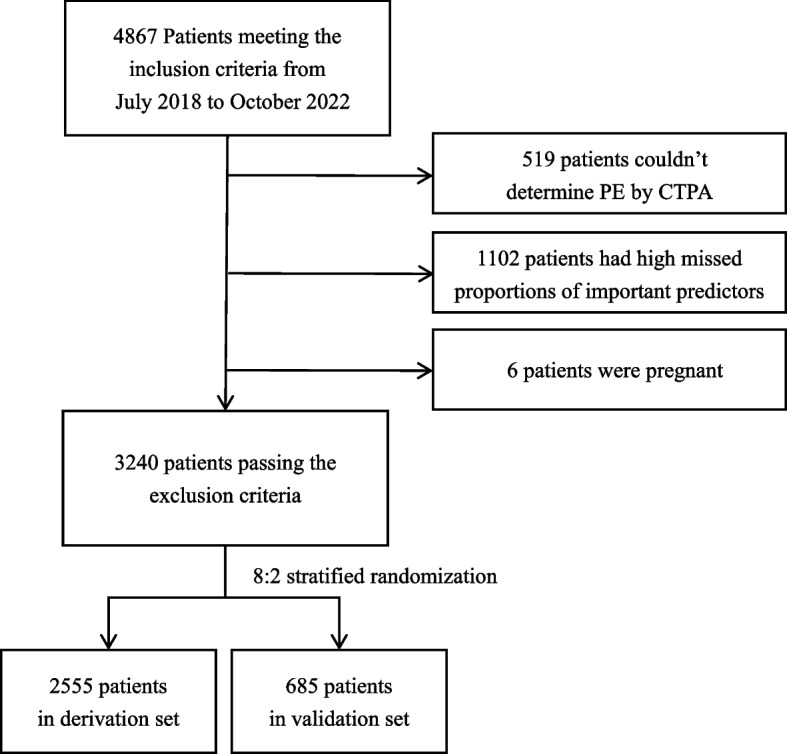
Study workflow. The workflow of excluding patients passing the exclusion criteria

A total of 4867 patients presenting to the emergency department of Zhongshan Hospital from July 2018 to October 2022 met the inclusion criteria and were included in the study. Among them, 519 could not be confirmed of PE status by CTPA, 1102 had high missing proportions of important predictors, 6 were pregnant and these patients were thus excluded from the study. Among 3240 patients included in the analysis, 2555 were randomly assigned to the derivation set and the remaining 685 patients were allocated to the validation set (Fig. 1). Baseline characteristics for patients in the derivation and validation set were described in Table 1. Overall, patients in the derivation and validation set were similar in terms of demographics, PE severity grade distribution, medical history, clinical signs and symptoms.

**Table 1:** Patient characteristics in the derivation and validation set

	**Derivation set (*n* = 2555)**	**Validation set (*n* = 685)**	***P* Value**
**Age, years, No. (%)**	68 (61—77)	68 (60—77)	0.58
**Sex, male, No. (%)**	1182 (46.3%)	312 (45.5%)	0.77
**PE severity grade, No. (%)**			
Severe	353 (13.8%)	88 (12.8%)	0.79
Moderate	140 (5.5%)	38 (5.5%)	
Mild	93 (3.6%)	21 (3.1%)	
No PE	1969 (77.1%)	538 (78.5%)	
**Symptoms, No. (%)**			
Anhelation	671 (26.3%)	186 (27.2%)	0.67
Lower limb edema	81 (3.2%)	31 (4.5%)	0.11
Chest distress	840 (32.9%)	214 (31.2%)	0.44
Chest pain	764 (29.9%)	210 (30.7%)	0.74
Hemoptysis	35 (1.4%)	9 (1.3%)	> 0.99
Syncope	188 (7.4%)	53 (7.7%)	0.80
Cough	60 (2.3%)	20 (2.9%)	0.47
Palpitation	248 (9.7%)	68 (9.9%)	0.92
**Signs, median (IQR)**			
Temperature, ℃	36.5 (36.2—36.8)	36.5 (36.2—36.7)	0.30
Heart rate, beats/min	88 (77—103)	88 (76—102)	0.91
Systolic blood pressure, mmHg	140 (122—158)	139 (120—156)	0.82
Diastolic blood pressure, mmHg	76 (66—86)	76 (67—86)	0.70
Oxygen saturation, %	96 (94—98)	96 (94—98)	0.67
**Past medical history, No. (%)**			
Cancer	86 (3.4%)	32 (4.7%)	0.13
Pulmonary embolism	61 (2.4%)	21 (3.1%)	0.39
Immobilization	584 (22.9%)	144 (21.0%)	0.33
Hypertension	423 (16.6%)	102 (14.9%)	0.15

Data are n (%), median (IQR), or mean (SD). Patients were randomly divided into the derivation and validation set using a stratified randomization scheme. P values were obtained using student t test for normally distributed variables, Wilcoxon rank sum test for skewed distributed variables and Chi-square (or Fisher’s exact) test for categorical variables

### Candidate predictors for PECSS

**Fig. 2:**
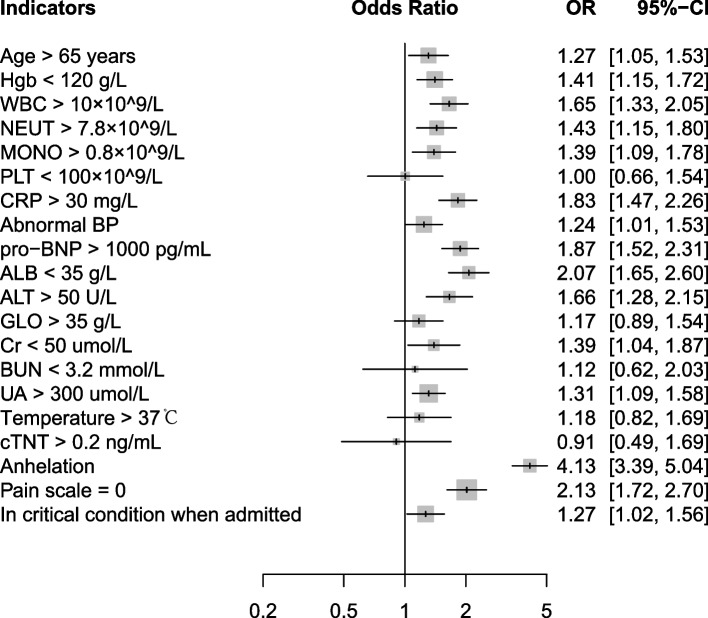
Univariate association of each clinical predictor with PE status. Odds ratio (OR) and 95% confidence interval (CI) were obtained from univariate logistic regression model. Hgb, hemoglobin; WBC, white blood cell count; NEUT, neutrophil count; MONO, monocyte count; PLT, platelet; CRP, c-reaction protein; abnormal BP, abnormal blood pressure; pro-BNP, N-terminal (NT)-pro hormone BNP; ALB, blood albumin; ALT, Alanine transaminase; GLO, globulin; Cr, creatinine; BUN, Blood Urea Nitrogen; UA, blood uric acid; cTNT, cardiac troponin test

Using data from 2555 patients in the derivation set, we assessed the association strength with PE status for each clinical predictor. Univariate logistic regression showed that age above 65 years old, low hemoglobin, high white blood cell, neutrophil and monocyte, high C-reaction protein, abnormal blood pressure, high N-terminal (NT)-pro hormone BNP, low blood albumin and high blood Alanine transaminase, low blood Creatinine, high blood Uric Acid, presence of anhelation, lower levels of pain scale and in critical condition when admitted were significantly associated with increased risk for PE (Fig. 2). Distribution of each candidate predictor and Wells score indicator across different PE severity grades in the derivation set can be found in Table S 1.

### PECSS scoring system

In addition to the six predictors already included in Wells score, seven candidate predictors (Anhelation, abnormal blood pressure, high pro-BNP, high CRP, in critical condition when admitted, Age > 65 years and high uric acid) selected by univariate logistic regression were also included in the searching algorithm to construct the optimal scoring system. Table 2 showed the optimal scoring system derived for each candidate predictor in addition to Wells score. PECSS was calculated as the sum of total scores obtained from all candidate predictors and Wells score indicators. To facilitate its application in clinical setting, we seek optimal PECSS cutoff values to group patients into different risk categories. Patients with PECSS no higher than 4 can be considered safe and exempt from CTPA. This is thereafter called PECSS only screening approach. Distribution of PECSS across different PE severity grades in the derivation set can be found in Figure S 1.

**Table 2:** Scoring system for candidate predictors and Wells score indicators in PECSS

Predictor	score
**Candidate predictor**	
Anhelation	1.5
Abnormal BP	1.0
pro-BNP > 1000 pg/mL	0.5
CRP > 30 mg/L	0.5
UA > 300 umol/L	0.5
In critical condition when admitted	0.5
Age > 65 years	0.5
**Wells score**	
VTE symptoms	3.0
No alternative diagnosis	3.0
HR > 100 beats/min	1.5
Immobilization/surgery	1.5
Previous PE/VTE	1.5
Hemoptysis	1.0
Active cancer	1.0

Abnormal blood pressure, defined as low blood pressure (systolic blood pressure < 90 mmHg or diastolic blood pressure < 60 mmHg) or high blood pressure (systolic blood pressure > 140 mmHg and diastolic blood pressure > 90 mmHg)

*VTE* Venous Thrombus Embolism, *HR* Heart Rate, *BP* Blood Pressure

### Incorporating D-Dimer with PECSS

**Fig. 3:**
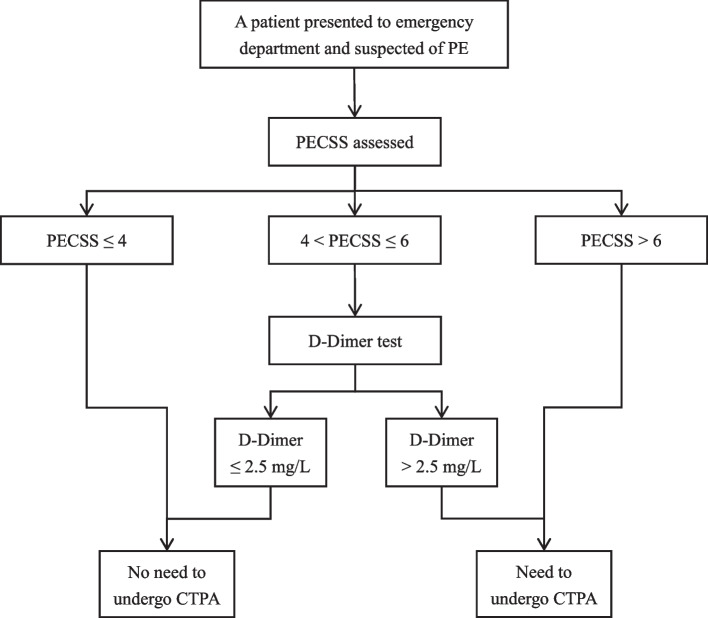
Flowchart summary of the PECSS + D-Dimer screening approach

Next, we incorporated D-Dimer with PECSS by seeking optimal D-Dimer cutoffs in each PECSS stratum. Distribution of D-Dimer across different PE severity grades and within each PECSS stratum can be found in Figure S 2 and Figure S 3. Decision framework of the PECSS + D-Dimer screening approach was summarized in Fig. 3. Specifically, for patients with PECSS no higher than 4, PE can be safely ruled out and no CTPA is needed; for patients with PECSS between 4 and 6, PE can only be safely ruled out among those with D-Dimer below 2.5 mg/L; for patients with PECSS higher than 6, CTPA should be routinely conducted and PE could not be safely ruled out.

### Comparison of screening performances in multiple scoring systems

We compared failure rates across all PE severity grades in multiple scoring systems: Wells score only, Wells score + D-Dimer, Wells_PERC, PECSS only, PECSS + D-Dimer and PECSS + PERC. Detailed description of each screening approach can be found in the Text S1. Failure rate decreased from 5.1% and 0.9% for Wells score and Wells score + D-Dimer approach, to 0.0% and 0.3% for PECSS and PECSS + D-Dimer approach among patients with severe PE. Similar trends were observed for the failure rates among patients with moderate, mild and any PE (Table 3). PCESS + PERC achieved zero failure rates across all PE severity grades, perfect sensitivity and specificity, which improved upon Wells + PERC. PECSS + PERC had the highest sensitivity, while Wells score and PECSS + D-Dimer had the highest specificity. PECSS + PERC had the highest NPV and Wells score had the highest PPV.

**Table 3:** Comparison of screening performances in four scoring system

	**Wells**	**Wells + D-Dimer**	**Wells + PERC**	**PECSS**	**PECSS + D-Dimer**	**PECSS + PERC**
**Primary endpoint**						
Failure rate in severe PE	5.1%	0.9%	0.0%	0.0%	0.3%	0.0%
**Secondary endpoints**						
Failure rate in moderate PE	16.5%	4.3%	0.0%	2.2%	4.3%	0.0%
Failure rate in mild PE	16.0%	6.4%	1.1%	0.0%	6.4%	0.0%
Failure rate in any PE	9.6%	2.9%	0.2%	0.5%	2.2%	0.0%
**Additional endpoints**						
Sensitivity	90.4%	97.1%	99.8%	99.5%	97.8%	100.0%
Specificity	66.7%	9.8%	7.4%	25.5%	52.0%	4.5%
NPV	95.9%	91.9%	99.3%	99.4%	98.7%	100.0%
PPV	44.9%	24.3%	24.3%	28.4%	37.7%	23.8%

*PPV* Positive predictive value, *NPV* Negative predictive value

### Validation of PECSS

**Fig. 4:**
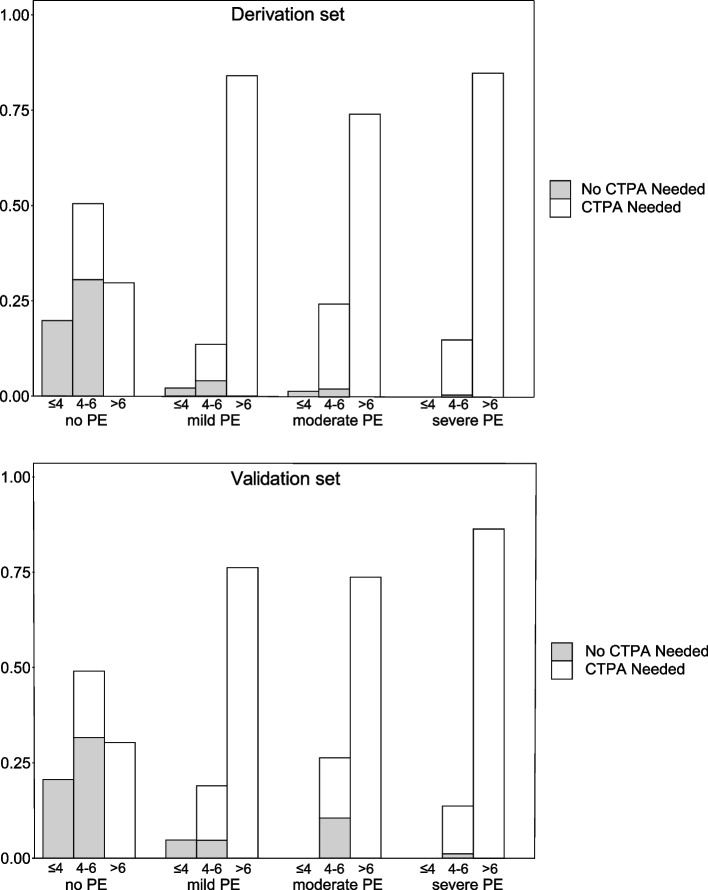
Distribution of PECSS among patients with different PE severity grades in the derivation and validation set. Shaded areas indicate patients proportions where PE can be safely ruled out and no CTPA is needed: PECSS no higher than 4, or 4 < PECSS ≤ 6 and D-Dimer ≤ 2.5 mg/L

Finally, results were validated using the independent held-out validation set. Distribution of each candidate predictor and Wells score indicator in the derivation set can be found in Table S 2. Similar to the derivation set, PECSS and PECSS + D-Dimer achieved lower failure rates among patients with severe, moderate, mild and any PE compared to Wells score and Wells + D-Dimer approaches in the validation set (Table S 3). Again, PECSS + PERC achieved zero failure rates across all PE severity grades. Distributions of PECSS and PECSS + D-Dimer across different PE severity grades were similar in the derivation and validation set, where higher PECSS and lower PE rule out rates were observed with patients with more severe PE (Fig. 4).

## Discussion

Due to the difficulties of PE diagnosis in early clinical stage and emergency medical setting [18, 19], physicians often rely on excessive CTPA use to reduce the miss diagnosis rate of PE, especially for those with pulmonary artery embolism or multiple pulmonary arteriole embolism, which could lead to poor prognosis and high risk of death. To rule out PE among patients presenting to the emergency department, the PERC [20] rule required 8 objective criteria, including age (less than 50 years old), medical history and several empirical indicator of clinical signs and symptoms [21]. However, with the aging population and potential assessment bias introduced by attending physicians of various clinical experiences in PE diagnosis, the application of PERC rule in emergency medical setting is relatively limited [22, 23].

In this investigation, we developed and validated an objective and easy-to-use scoring system to safely rule out PE among patients presenting to the emergency department. The newly proposed Pulmonary Embolism Comprehensive Screening Score (PECSS) combined important clinical predictors with indicators in the Wells score and achieved excellent performances across all PE severity grades. Particularly, we found that patients with age > 65 years old, anhelation, less pain, abnormal blood pressure, in critical condition when admitted, higher levels of pro-BNP, CRP, UA, WBC, NEUT, MONO and ALT, and lower levels of HGB, ALB and creatinine were at increased risk for PE. Through a comprehensive grid searching algorithm, PECSS identified 7 clinical predictors (anhelation, abnormal blood pressure, pro-BNP > 1000 pg/mL, CRP > 30 mg/L, UA > 300 umol/L, in critical condition when admitted and age > 65 years), in addition to indicators in Wells score (VTE symptoms, no alternative diagnosis, HR > 100 beats/min, immobilication / surgery and previous PE/VTE), and assigned corresponding scores to each of the predictor. All of the clinical predictors were easy to obtain from medical records and rountine blood tests in emergency department setting.

Identification of these candidate clinical predictors were supported by previous studies. Conditions such as pulmonary main embolism can lead to obstructive shock, which is a possible cause of hypotension. Elevated blood pressure can be explained by PE associated symptoms or stress [24, 25]. Although a large number of studies have found pro-BNP as a biomarker for PE, optimal cut-off values are still inconsistent [26–28]. In our study, we assigned 0.5 point to pro-BNP greater than 1000 ng/L in constructing PECSS and provided a reference for future research regarding the effect of pro-BNP on PE. Both CRP and UA are acute phase reactant, which are associated with increased risk of cardiovascular and cerebrovascular events [29–33]. CRP has been shown to increase the tendency to thrombose through the mediation of interleukin 6 and monocyte chemoattractant protein 1 [34, 35]. Plasma level of UA can be used as a risk marker for thrombosis and prognostic stratification among PE patients [36, 37]. Therapies targeted at lowering UA are associated with reduced risk of major adverse cardiovascular events [38–40].

To facilitate clinical application and further improve the screening performance, we incorporated plasma level of D-Dimer, which is often elevated in the presence of acute thrombosis, with PECSS. Optimal D-Dimer cutoff values were investigated in patients with low (PECSS $\le$ 4), moderate (4 < PECSS $\le$ 6) and high (PECSS > 6) PE risk. It turned out that D-Dimer only played a role in patients with moderate PE risk, where higher levels of plasma D-Dimer increased the pretest probability of PE. Therefore, patients with moderate risk (4 < PECSS $\le$ 6) and low D-Dimer (D-Dimer $\le$ 2.5 mg/L) could be safely ruled out of PE, whereas those with moderate risk (4 < PECSS $\le$ 6) and high D-Dimer (D-Dimer > 2.5 mg/L) need to undergo CTPA before PE can be ruled out. D-Dimer were not expected to affect the decision of ruling out PE in patients with low and high pretest probability, since patients with low PECSS have very low risk of PE according to medical records and clinical symptoms where D-Dimer do not have enough clinical specificity, while those with high PECSS have high risk of PE such that they need to undergo CTPA regardless of their plasma D-Dimer level.

Screening performances of PECSS only, PECSS + D-Dimer, Wells only, Wells + D-Dimer were thoroughly compared in terms of failure rates across all PE severity grades, sensitivity, specificity, PPV and NPV. Compared to Wells only, PECSS only approach greatly reduced the failure rates in all PE severity grades (5.1% to 0, 16.5% to 2.2%, 16.0% to 0, and 9.6% to 0.5% in severe, moderate, mild and any PE, respectively). The huge improvement indicated that by including the additional seven clinical predictors chosen by variable selection and clinical meaningfulness, PECSS are able to identify patients with suspected PE risk more accurately and efficiently than Wells score. Although PECSS + D-Dimer and Wells + D-Dimer had similar performances in moderate and mild PE, PECSS + D-Dimer had three times lower failure rate in severe PE compared to Wells + D-Dimer (0.3% versus 0.9%). This is of particular clinical significance since servere PE consists of patients with pulmonary artery embolism or multiple pulmonary arteriole embolism and had the worst clinical prognosis [41–44]. By improving the accuracy and effectiveness of pretest probability and targeting on the clinically beneficial patient population, the PECSS + D-Dimer would greatly reduce the mortality associated with PE. In terms of sensitivity and specificity, Wells score achieved the highest specificity, but at the cost of low sensitivity and NPV. PECSS had the highest sensitivity, while at the cost of low specificity and PPV. PECSS + D-Dimer had the best compromise between good sensitivity and specificity, and moderately high PPV and NPV. It should be noted that although PECSS had very high sensitivity, its specificity and positive predictive value were relatively low. This indicates that PECSS works well in identifying patients at high risk of PE, but relatively under-performs to rule out PE when patients are screened negative. PECSS + D-Dimer strategy improved upon PECSS only in terms of both specificity and positive predictive value.

Admittedly, our study has several limitations. Firstly, this is a single center study and generalization of the results require validation from multi-center set. Secondly, patients who were suspected of PE but did not undergo CTPA due to hemodynamic instability were not included in out study due to the uncertainty of their PE status. This would potentially lead to bias in applying PECSS and PECSS + D-Dimer approaches in the targeted patient population. Thirdly, radiological severity instead of clinical severity (such as hypotension, pulmonary hypertension, cardiac failure, and the need for oxygen therapy) were used when defining PE severity grades. However, they do not usually correlate perfectly with each other. Further, right ventricular dysfunction was not considered in defining the PE severity grades since echocardiography was missing for a large proportion of the study population. Although CTPA has been applied in right ventricular dysfunction, its reliability and accuracy cannot be guaranteed as echocardiography [17, 18]. Finally, our study is retrospective in nature, and therefore, causal relationships could not be established between clinical predictors in constructing PECSS and PE diagnosis.

## Conclusions

In conclusion, in this study we developed a new pretest probability (PECSS) that is more comprehensive and accurate than the well established Wells score for PE diagnosis, and incorporated plasma level of D-Dimer with PECSS as a clinical instrument to rule out PE among suspected patients presenting to the emergency department. Both PECSS and PECSS + D-Dimer approaches showed excellent performances in reducing PE cases that were ruled out, and the advantages over Wells score and Wells + D-Dimer approaches were more pronounced among patients with severe PE, which would usually lead to worst prognosis and highest mortality. With PECSS and D-Dimer, PE diagnosis could be operated more efficiently without overuse of CTPA. Finally, all clinical predictors in PECSS are easy to obtain from medical records and routine blood tests, making PECSS a practical tool in emergency medical setting.

### Supplementary Information

**Additional file 1: Text S1.** Detailed description of four scoring systems. **Figure S1.** Distribution of PECSS across different PE severity grades in the derivation set. **Figure S2.** Distribution of D-Dimer across different PE severity grades in the derivation set. **Figure S3.** Distribution of D-Dimer within each PECSS stratum in the derivation set. **Table S1.** Distribution of each candidate predictor and Well’s score indicator across different PE severity grades in the derivation set.

## Data Availability

Data and materials will be available from the corresponding authors upon reasonable request.

## Abbreviations

PECSS: Pulmonary Embolism Comprehensive Screening Score
PE: Pulmonary embolism
CTPA: Computed tomographic pulmonary angiography
OR: Odds ratios
CI: Confidence interval
Hgb: Hemoglobin
WBC: White blood cell count
NEUT: Neutrophil count
MONO: Monocyte count
PLT: Platelet
CRP: C-reaction protein
abnormal BP: Abnormal blood pressure
pro-BNP: N-terminal (NT)-pro hormone BNP
ALB: Blood albumin
ALT: Alanine transaminase
GLO: Globulin
Cr: Creatinine
BUN: Blood Urea Nitrogen
UA: Blood uric acid
cTNT: Cardiac troponin test
PPV: Positive predictive value
NPV: Negative predictive value
VTE: Venous Thrombus Embolism
HR: Heart Rate
BP: Blood Pressure

## References

[1] Jiménez D, de Miguel-Díez J, Guijarro R (2016). Trends in the Management and Outcomes of Acute Pulmonary Embolism: Analysis From the RIETE Registry. J Am Coll Cardiol.

[2] Kahn SR, de Wit K (2022). Pulmonary Embolism. N Engl J Med.

[3] KKeller K, Hobohm L, Ebner M (2020). Trends in thrombolytic treatment and outcomes of acute pulmonary embolism in Germany. Eur Heart J..

[4] Douma RA, Mos IC, Erkens PM (2011). Performance of 4 clinical decision rules in the diagnostic management of acute pulmonary embolism: a prospective cohort study. Ann Intern Med.

[5] Pollack CV, Schreiber D, Goldhaber SZ (2011). Clinical characteristics, management, and outcomes of patients diagnosed with acute pulmonary embolism in the emergency department: initial report of EMPEROR (Multicenter Emergency Medicine Pulmonary Embolism in the Real World Registry). J Am Coll Cardiol.

[6] Konstantinides SV, Meyer G (2019). The 2019 ESC guidelines on the diagnosis and management of acute pulmonary embolism. Eur Heart J.

[7] Konstantinides SV, Torbicki A, Agnelli G, et al. 2014 ESC guidelines on the diagnosis and management of acute pulmonary embolism [published correction appears in Eur Heart J. 2015 Oct 14;36(39):2666] [published correction appears in Eur Heart J. 2015 Oct 14;36(39):2642]. Eur Heart J. 2014;35(43):3033–3069k. 10.1093/eurheartj/ehu283. 10.1093/eurheartj/ehu283 25173341

[8] Kline JA, Garrett JS, Sarmiento EJ, Strachan CC, Courtney DM (2020). Over-testing for suspected pulmonary embolism in american emergency departments: the continuing epidemic. Circ Cardiovasc Qual Outcomes.

[9] Konstantinides SV, Barco S, Lankeit M, Meyer G (2016). Management of pulmonary embolism: an update. J Am Coll Cardiol.

[10] Le Gal G, Righini M, Roy PM (2006). Prediction of pulmonary embolism in the emergency department: the revised Geneva score. Ann Intern Med.

[11] Wells PS, Anderson DR, Bormanis J (1997). Value of assessment of pretest probability of deep-vein thrombosis in clinical management. Lancet.

[12] Kohn MA, Klok FA, van Es N (2017). D-dimer interval likelihood ratios for pulmonary embolism. Acad Emerg Med.

[13] Linkins LA, Bates SM, Ginsberg JS, Kearon C (2004). Use of different D-dimer levels to exclude venous thromboembolism depending on clinical pretest probability. J Thromb Haemost.

[14] Kearon C, de Wit K, Parpia S (2019). Diagnosis of pulmonary embolism with d-Dimer adjusted to clinical probability. N Engl J Med.

[15] Righini M, Kamphuisen PW, Le Gal G (2014). Age-adjusted D-dimer cutoff levels and pulmonary embolism–reply. JAMA.

[16] van Es N, van der Hulle T, van Es J (2016). Wells rule and d-Dimer testing to rule out pulmonary embolism: a systematic review and individual-patient data meta-analysis. Ann Intern Med.

[17] Barrios D, Morillo R, Yusen RD, Jiménez D (2018). Pulmonary embolism severity assessment and prognostication. Thromb Res.

[18] Cohen AT, Agnelli G, Anderson FA (2007). Venous thromboembolism (VTE) in Europe. The number of VTE events and associated morbidity and mortality. Thromb Haemost..

[19] Stein PD, Henry JW (1995). Prevalence of acute pulmonary embolism among patients in a general hospital and at autopsy. Chest.

[20] Kline JA, Mitchell AM, Kabrhel C, Richman PB, Courtney DM (2004). Clinical criteria to prevent unnecessary diagnostic testing in emergency department patients with suspected pulmonary embolism. J Thromb Haemost.

[21] Penaloza A, Soulié C, Moumneh T (2017). Pulmonary embolism rule-out criteria (PERC) rule in European patients with low implicit clinical probability (PERCEPIC): a multicentre, prospective, observational study. Lancet Haematol.

[22] Liang X, Gao W, Li S. Epidemiological study of 270003 cases of Grade I to III emergency patients. J Clin Emerg.

[23] Ambrosetti E (2022). Europe: Low Fertility, Aging, and Migration Policies.

[24] Keller K, Beule J, Balzer JO, Dippold W (2015). Blood pressure for outcome prediction and risk stratification in acute pulmonary embolism. Am J Emerg Med.

[25] Konstam MA, Kiernan MS, Bernstein D (2018). Evaluation and management of right-sided heart failure: a scientific statement from the American Heart Association. Circulation.

[26] Jiménez D, Kopecna D, Tapson V (2014). Derivation and validation of multimarker prognostication for normotensive patients with acute symptomatic pulmonary embolism. Am J Respir Crit Care Med.

[27] den Exter PL, Zondag W, Klok FA (2016). Efficacy and safety of outpatient treatment based on the hestia clinical decision rule with or without N-Terminal pro-brain natriuretic peptide testing in patients with acute pulmonary embolism. a randomized clinical trial. Am J Respir Crit Care Med.

[28] Binder L, Pieske B, Olschewski M (2005). N-terminal pro-brain natriuretic peptide or troponin testing followed by echocardiography for risk stratification of acute pulmonary embolism. Circulation.

[29] Ozsu S, Yilmaz G, Yilmaz I, Oztuna F, Bulbul Y, Ozlu T (2011). C-reactive protein alone or combined with cardiac troponin T for risk stratification of respiratory intensive care unit patients. Respir Care.

[30] Ridker PM (1998). C-reactive protein and risks of future myocardial infarction and thrombotic stroke. Eur Heart J.

[31] Ridker PM, Buring JE, Shih J, Matias M, Hennekens CH (1998). Prospective study of C-reactive protein and the risk of future cardiovascular events among apparently healthy women. Circulation.

[32] Ioachimescu AG, Brennan DM, Hoar BM, Hazen SL, Hoogwerf BJ (2008). Serum uric acid is an independent predictor of all-cause mortality in patients at high risk of cardiovascular disease: a preventive cardiology information system (PreCIS) database cohort study. Arthritis Rheum.

[33] Warwick G, Thomas PS, Yates DH (2008). Biomarkers in pulmonary hypertension. Eur Respir J.

[34] Verma S, Yeh ET (2003). C-reactive protein and atherothrombosis–beyond a biomarker: an actual partaker of lesion formation. Am J Physiol Regul Integr Comp Physiol.

[35] Pasceri V, Willerson JT, Yeh ET (2000). Direct proinflammatory effect of C-reactive protein on human endothelial cells. Circulation.

[36] Shimizu Y, Nagaya N, Satoh T (2002). Serum uric acid level increases in proportion to the severity of pulmonary thromboembolism. Circ J.

[37] Ozsu S, Çoşar AM, Aksoy HB (2017). Prognostic value of uric acid for pulmonary thromboembolism. Respir Care.

[38] Gill D, Cameron AC, Burgess S (2021). Urate, blood pressure, and cardiovascular disease: evidence from mendelian randomization and meta-analysis of clinical trials. Hypertension.

[39] Țăpoi L, Șalaru DL, Sascău R, Stătescu C (2021). Uric Acid-An Emergent Risk Marker for Thrombosis?. J Clin Med..

[40] Bartziokas K, Kyriakopoulos C, Potonos D, Exarchos K, Gogali A, Kostikas K (2022). The Diagnostic Role of Uric Acid to Creatinine Ratio for the Identification of Patients with Adverse Pulmonary Embolism Outcomes. Diagnostics (Basel)..

[41] Irmak I, Sertçelik Ü, Öncel A (2020). Correlation of thrombosed vessel location and clot burden score with severity of disease and risk stratification in patients with acute pulmonary embolism. Anatol J Cardiol.

[42] Vedovati MC, Germini F, Agnelli G, Becattini C (2013). Prognostic role of embolic burden assessed at computed tomography angiography in patients with acute pulmonary embolism: systematic review and meta-analysis. J Thromb Haemost.

[43] Ghanima W, Abdelnoor M, Holmen LO, Nielssen BE, Sandset PM (2007). The association between the proximal extension of the clot and the severity of pulmonary embolism (PE): a proposal for a new radiological score for PE. J Intern Med.

[44] Shen C, Yu N, Wen L (2019). Risk stratification of acute pulmonary embolism based on the clot volume and right ventricular dysfunction on CT pulmonary angiography. Clin Respir J.

