# Supplementary material for: PECSS: Pulmonary Embolism Comprehensive Screening Score to safely rule out pulmonary embolism among suspected patients presenting to emergency department
Source: BMC Pulm Med. 2023 Aug 7;23:287. doi: 10.1186/s12890-023-02580-8 (PMC10408070; doi:10.1186/s12890-023-02580-8)
Supplement: Supplementary file 1 — Additional file 1: Text S1. Detailed description of four scoring systems. Figure S1. Distribution of PECSS across different PE severity grades in the derivation set. Figure S2. Distribution of D-Dimer across different PE severity grades in the derivation set. Figure S3. Distribution of D-Dimer within each PECSS stratum in the derivation set. Table S1. Distribution of each candidate predictor and Well’s score indicator across different PE severity grades in the derivation set. [file 12890_2023_2580_MOESM1_ESM.docx]

| **Numbered pages** | **Contents** |
| --- | --- |
| Page 2 | Text S1. Detailed description of four scoring systems. |
| Page 3 | Figure S1. Distribution of PECSS across different PE severity grades in the derivation set. |
| Page 4 | Figure S2. Distribution of D-Dimer across different PE severity grades in the derivation set. |
| Page 5 | Figure S3. Distribution of D-Dimer within each PECSS stratum in the derivation set. |
| Page 6 | Table S1. Distribution of each candidate predictor and Well’s score indicator across different PE severity grades in the derivation set. |
| Page 7 | Table S2. Distribution of each candidate predictor and Well’s score indicator across different PE severity grades in the validation set. |
| Page 8 | Table S3. Comparison of screening performances in four scoring system in the validation set. |
| Page9 | Reference |

**Text S1. Detailed description of four scoring systems**

**Wells score only:**

If Wells score ≤ 4, patients were diagnosed as no PE and other patients needed to undergo CTPA.^1^

**Wells score + D-Dimer:**

If Wells score ≤ 4 and D-Dimer ≤ 1 mg/L, patients were diagnosed as no PE. If 4 < Wells score ≤ 6 and D-Dimer ≤ 0.5 mg/L, patients were diagnosed as no PE. Other patients needed to undergo CTPA.^2^

**PECSS only:**

If PECSS ≤ 4, patients were diagnosed as no PE and other patients needed to undergo CTPA.

**PECSS + D-Dimer:**

If PECSS ≤ 4, patients were diagnosed as no PE. If 4 < PECSS ≤ 6 and D-Dimer ≤ 2.5 mg/L, patients were diagnosed as no PE. Other patients needed to undergo CTPA.


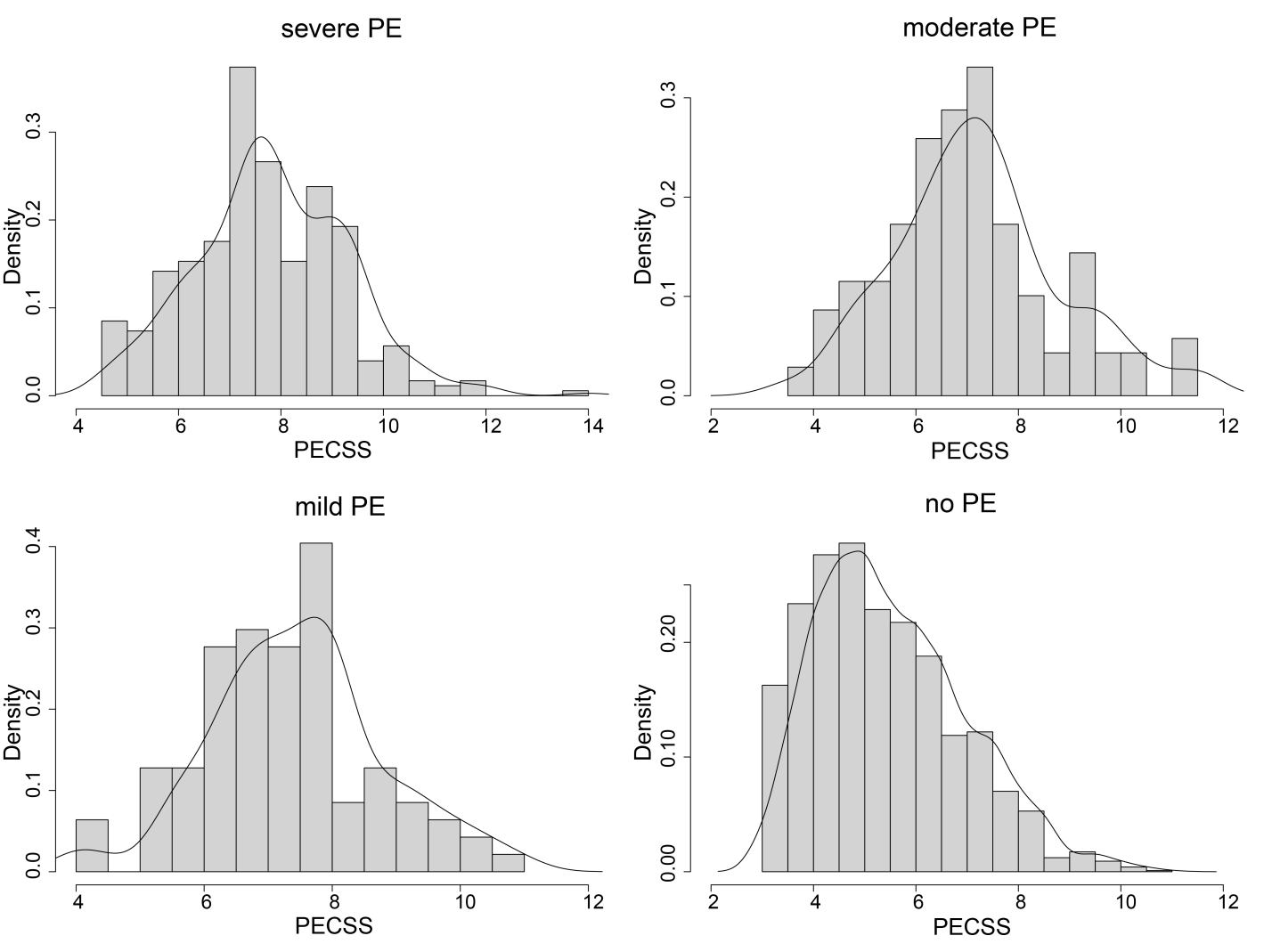


**Figure S1. Distribution of PECSS across different PE severity grades in the derivation set**


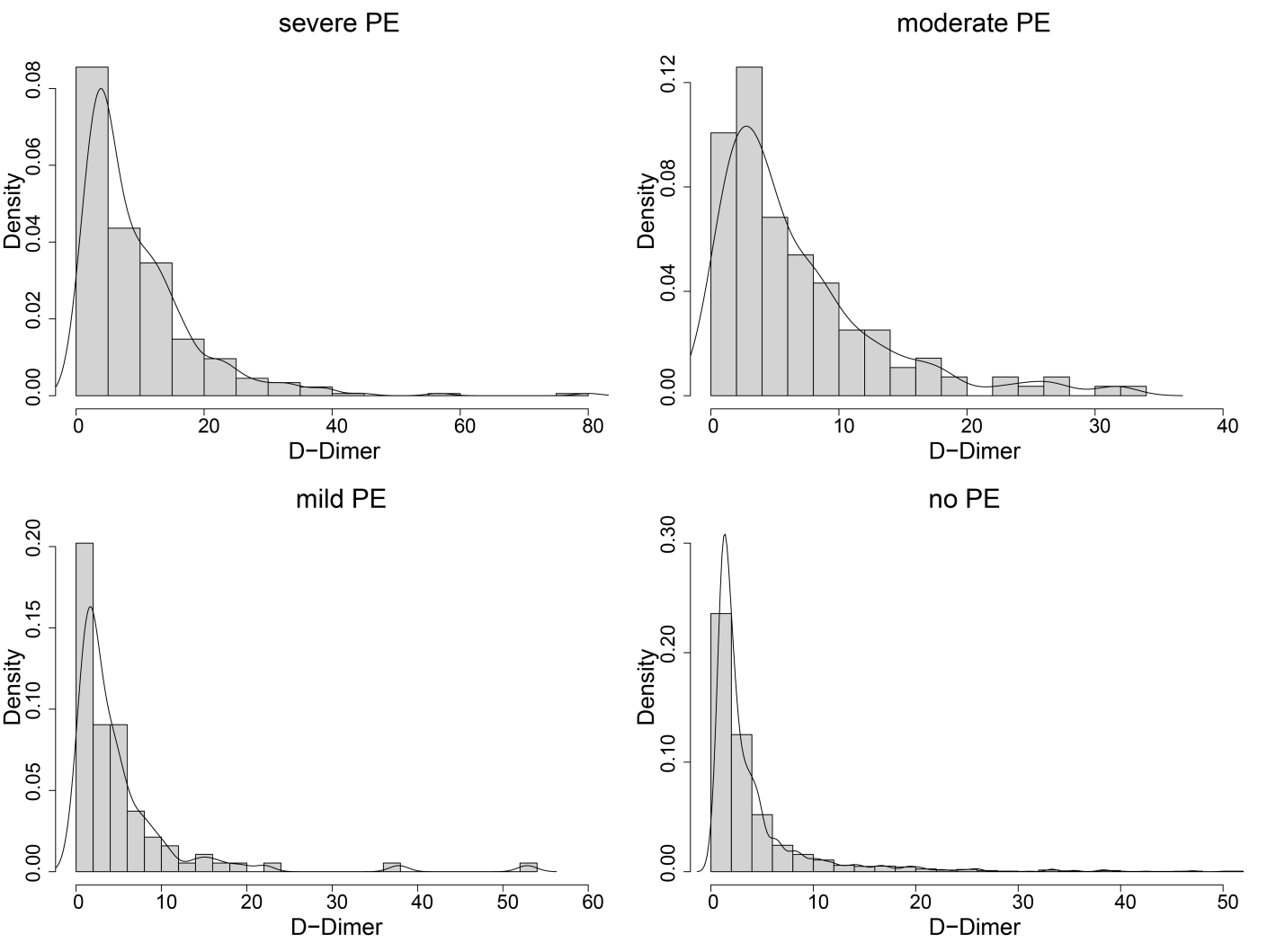


**Figure S2. Distribution of D-Dimer across different PE severity grades in the derivation set**


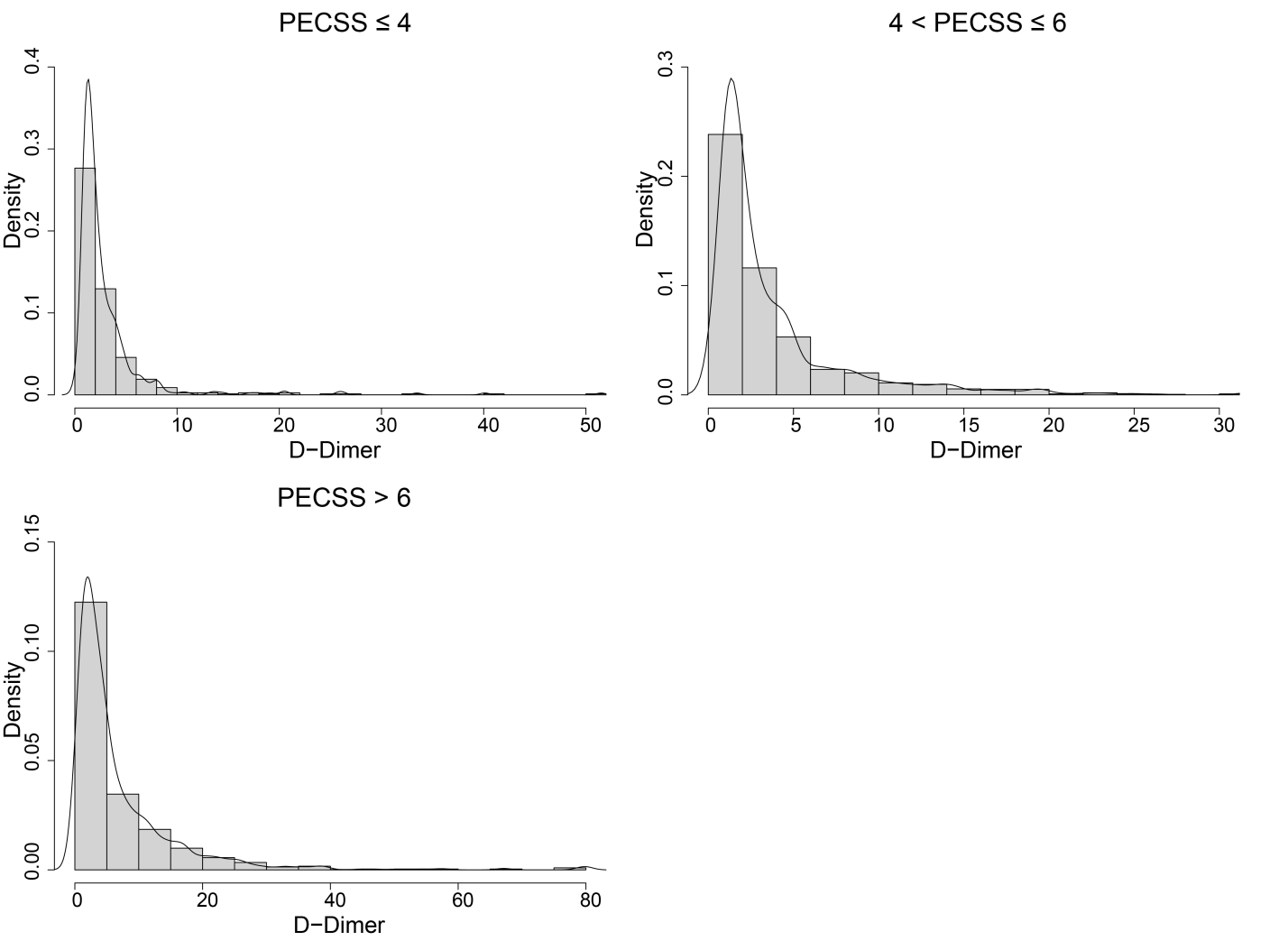


**Figure S3. Distribution of D-Dimer within each PECSS stratum in the derivation set**

| **Table S1. Distribution of each candidate predictor and Well’s score indicator across different PE severity grades in the derivation set** | | | | | | |
| --- | --- | --- | --- | --- | --- | --- |
|  | **all**  **(n=2555)** | **severe PE**  **(n=353)** | **moderate PE**  **(n=139)** | **mild PE**  **(n=94)** | **no PE**  **(n=1969)** | **P Value** |
| **Wells indicators****, No. (%)** |  |  |  |  |  |  |
| VTE symptoms | 83 (3.2%) | 16 (4.5%) | 6 (4.3%) | 8 (8.5%) | 53 (2.6%) | 0.01 |
| No alternative diagnosis | 2555 (100.0%) | 353 (100.0%) | 139 (100.0%) | 94 (100.0%) | 1969 (100.0%) | > 0.99 |
| HR >100 beats/min | 715 (27.9%) | 145 (41.0%) | 41(29.4%) | 28 (29.7%) | 501 (25.4%) | < 0.001 |
| Immobilization/surgery | 585 (22.8%) | 307 (86.9%) | 102 (73.3%) | 65 (69.1%) | 111 (5.6%) | < 0.001 |
| Previous PE/VTE | 61 (2.3%) | 17 (4.8%) | 7 (5.0%) | 4 (4.2%) | 33 (1.6%) | < 0.001 |
| Hemoptysis | 36 (1.4%) | 6 (1.6%) | 2 (1.4%) | 3 (3.2%) | 25 (1.2%) | 0.45 |
| Active cancer | 215 (8.4%) | 56 (15.8%) | 22 (15.8%) | 11 (11.7%) | 126 (6.3%) | < 0.001 |
| **Candidate indicators, No. (%)** |  |  |  |  |  |  |
| Anhelation | 1118 (43.7%) | 263 (76.5%) | 86 (61.8%) | 63 (67.0%) | 706 (35.8%) | <0.001 |
| Abnormal BP | 725 (28.3%) | 90 (25.4%) | 42 (30.2%) | 33 (35.1%) | 560 (28.4%) | 0.29 |
| pro-BNP > 1000 pg/mL | 560 (21.9%) | 117 (33.1%) | 37 (26.6%) | 27 (28.7%) | 379 (19.2%) | < 0.001 |
| CRP > 30 mg/L | 506 (19.8%) | 103 (29.2%) | 37 (26.6%) | 23 (24.5%) | 343 (17.4%) | < 0.001 |
| UA > 300 umol/L | 1139 (44.5%) | 180 (50.9%) | 65 (46.7%) | 46 (48.9%) | 848 (43.0%) | 0.03 |
| In critical condition when admitted | 1837 (71.8%) | 274 (77.6%) | 99 (71.2%) | 70 (74.5%) | 1394 (70.7%) | 0.06 |
| Age > 65 years | 1530 (59.9%) | 200 (56.7%) | 89 (64.0%) | 65 (69.1%) | 1176 (59.7%) | 0.12 |
| **PECSS, median (IQR)** |  |  |  |  |  |  |
| PECSS score | 6.0 (4.5 - 7.0) | 7.5 (7.0 - 9.0) | 7.0 (6.0 - 8.0) | 7.5 (6.5 - 8.0) | 5.5 (4.5 - 6.5) | < 0.001 |
| p values were obtained by Chi-square tests for categorical variables and ANOVA for continuous variables. | | | | | | |

| **Table S2. Distribution of each candidate predictor and Well’s score indicator across different PE severity grades in the validation set** | | | | | | |
| --- | --- | --- | --- | --- | --- | --- |
|  | **all**  **(n=685)** | **severe PE**  **(n=88)** | **moderate PE**  **(n=38)** | **mild PE**  **(n=21)** | **no PE**  **(n=538)** | **P Value** |
| **Wells indicators, No. (%)** |  |  |  |  |  |  |
| VTE symptoms | 31 (4.5%) | 4 (4.5%) | 3 (7.8%) | 2 (9.5%) | 20 (3.7%) | 0.01 |
| No alternative diagnosis | 685 (100.0%) | 88 (100.0%) | 38 (100.0%) | 21 (100.0%) | 538 (100.0%) | > 0.99 |
| HR >100 beats/min | 188 (27.4%) | 43 (48.8%) | 16 (42.1%) | 5 (23.8%) | 124 (23.0%) | < 0.001 |
| Immobilization/surgery | 146 (21.3%) | 71 (80.6%) | 28 (73.6%) | 13 (61.9%) | 34 (6.3%) | < 0.001 |
| Previous PE/VTE | 22 (3.2%) | 11 (12.5%) | 0 (0.0%) | 2 (9.5%) | 9 (1.6%) | < 0.001 |
| Hemoptysis | 9 (1.3%) | 2 (2.2%) | 2 (5.2%) | 1 (4.7%) | 4 (0.7%) | 0.04 |
| Active cancer | 67 (9.7%) | 14 (15.9%) | 8 (21.0%) | 4 (19.0%) | 41 (7.6%) | 0.01 |
| **Candidate indicators, No. (%)** |  |  |  |  |  |  |
| Anhelation | 292 (42.6%) | 60 (68.1%) | 23 (60.5%) | 13 (61.9%) | 196 (36.4%) | < 0.001 |
| Abnormal BP | 171 (24.9%) | 23 (26.1%) | 14 (36.8%) | 6 (28.5%) | 128 (23.7%) | 0.33 |
| pro-BNP > 1000 pg/mL | 127 (18.5%) | 31 (35.2%) | 9 (23.6%) | 5 (23.8%) | 82 (15.2%) | < 0.001 |
| CRP > 30 mg/L | 153 (22.3%) | 30 (34.1%) | 13 (34.2%) | 1 (4.8%) | 109 (20.3%) | 0.01 |
| UA > 300 umol/L | 319 (46.5%) | 56 (63.6%) | 22 (57.8%) | 10 (47.6%) | 231 (42.9%) | 0.01 |
| In critical condition when admitted | 495 (72.2%) | 67 (76.1%) | 28 (73.6%) | 13 (61.9%) | 387 (71.9%) | 0.60 |
| Age > 65 years | 405 (59.1%) | 48 (54.5%) | 25 (65.8%) | 17 (80.9%) | 315 (58.6%) | 0.13 |
| **PECSS, median (IQR)** |  |  |  |  |  |  |
| PECSS score | 6 (4.5 - 7.0) | 8 (7.0 - 9.0) | 7 (6.0 - 8.5) | 7.5 (6.5 - 8.5) | 5.5 (4.5 - 6.5) | < 0.001 |

| **Table S3. Comparison of screening performances in four scoring system in the validation set** | | | | | | |
| --- | --- | --- | --- | --- | --- | --- |
|  | **Wells** | **Wells + D-Dimer** | **Wells + PERC** | **PECSS** | **PECSS + D-Dimer** | **PECSS+ PERC** |
| **Primary endpoint** |  |  |  |  |  |  |
| Failure rate in severe PE | 8.0% | 1.1% | 0.0% | 0.0% | 1.1% | 0.0% |
| **Secondary endpoints** |  |  |  |  |  |  |
| Failure rate in moderate PE | 15.8% | 4.3% | 2.6% | 0.0% | 2.6% | 0.0% |
| Failure rate in mild PE | 14.3% | 6.4% | 0.0% | 4.8% | 9.5% | 0.0% |
| Failure rate in any PE | 10.9% | 2.7% | 0.7% | 0.7% | 2.7% | 0.0% |
| **Additional endpoints** |  |  |  |  |  |  |
| Sensitivity | 89.1% | 97.3% | 99.3% | 99.3% | 97.3% | 100.0% |
| Specificity | 68.8% | 11.9% | 5.6% | 26.2% | 54.1% | 3.7% |
| NPV | 95.9% | 94.1% | 96.8% | 99.2% | 98.6% | 100.0% |
| PPV | 43.8% | 23.2% | 22.3% | 26.9% | 36.7% | 22.1% |
| PPV, positive predictive value; NPV, negative predictive value. | | | | | | |

**References**

1. Wells PS, Anderson DR, Bormanis J, et al. Value of assessment of pretest probability of deep-vein thrombosis in clinical management. The Lancet. 1997;350(9094):1795-8.
2. Kearon C, de Wit K, Parpia S, et al. Diagnosis of pulmonary embolism with d-dimer adjusted to clinical probability. New England Journal of Medicine. 2019 Nov 28;381(22):2125-34.
